# Clinical Management of Ovarian Hyperstimulation Syndrome with Furosemide at a Tertiary Referral Center—A Retrospective Data Analysis

**DOI:** 10.3390/jcm15166468

**Published:** 2026-08-21

**Authors:** Daniel Mayrhofer, Katharina Walch, Marlene Hager, Rebecca Künz, Alina Hoeltz, Rodrig Marculescu, Johannes Ott, Julian Marschalek

**Affiliations:** 1 Department of Obstetrics and Gynaecology, Clinical Division of Gynaecologic Endocrinology and Reproductive Medicine, Medical University of Vienna, 1090 Vienna, Austria; daniel.mayrhofer@meduniwien.ac.at (D.M.); katharina.walch@meduniwien.ac.at (K.W.); marlene.hager@meduniwien.ac.at (M.H.); n11932657@students.meduniwien.ac.at (R.K.); n11838189@students.meduniwien.ac.at (A.H.); julian.marschalek@meduniwien.ac.at (J.M.); 2 Department of Laboratory Medicine, Medical University of Vienna, 1090 Vienna, Austria; rodrig.marculescu@medunwien.ac.at

**Keywords:** ovarian hyperstimulation syndrome, OHSS, controlled ovarian stimulation, furosemide

## Abstract

**Background/Objective:** Ovarian hyperstimulation syndrome (OHSS) is a severe complication during controlled ovarian hyperstimulation. Ascites and consecutive hemoconcentration may lead to potentially life-threatening complications like thromboembolic events and the need for intensive care. The use of diuretics is widely used to treat ascites in non-OHSS patients. The aim of this study was to assess whether the use of furosemide in the treatment of OHSS was associated with a higher rate of complications compared to the literature. **Methods:** In this retrospective cohort study, 502 cases treated from January 2005 to June 2023 for OHSS at the Medical University of Vienna were evaluated regarding the distribution of OHSS severity, average duration of treatment, types and frequency of complications associated with antidiuretic treatment, and predictive factors for paracentesis. **Results:** The median AMH levels were 5.7 ng/mL. An antagonist protocol was used in 80% of stimulations. Human Chorionic Gonadotropin trigger was used in 80% of stimulations, with 71.7% of cases being early-onset. The incidence dropped from 8.4% (2006 to 2016) to 3.3% (from 2015 to 2023). The median duration of inpatient treatment was seven days, with about 75% of patients receiving furosemide as a diuretic treatment. Ascites drainage was necessary in 27.9%. None of our patients developed a thromboembolic complication following the furosemide treatment. **Conclusions:** During the study period, the incidence of OHSS declined, most probably as a result of advances in ovarian stimulation protocols and freezing strategies. In moderate to severe OHSS, standardized diuretic treatment with furosemide was not associated with additional thromboembolic complications as long as patients remain normovolemic.

## 1. Introduction

Ovarian hyperstimulation syndrome (OHSS) is an iatrogenic and potentially life-threatening complication during controlled ovarian hyperstimulation (COH) in the course of assisted reproductive technologies (ART). With an estimated prevalence of 1% to 5% for moderate-to-severe forms, it is considered a relatively rare condition [1]. However, with an increasing demand for ART, the management of OHSS, especially of more severe forms, still poses challenges to emergency clinicians, gynecologists, and reproductive specialists. As important pathophysiologic pathways behind the development of OHSS have been discovered, several risk factors have been determined. Moreover, improvements in reproductive technologies, namely the introduction of the antagonist protocol, the use of gonadotropin-releasing hormone agonists (GnRHa) as a trigger for oocyte maturation, as well as progress in embryo vitrification, have led to a decrease in the occurrence of OHSS [1,2,3]. Since fertility preservation (FP), especially in oncology patients, has become a new focus of reproductive specialists, maximizing mature oocyte yield within a limited time frame before induction of gonadotoxic treatments is frequently required. A careful balance must be struck between achieving a high oocyte yield, often within a single stimulation cycle, and ensuring patient safety, as moderate or severe OHSS may further delay cancer treatment. Consequently, optimization of OHSS management remains clinically relevant despite major advances in prevention.

Main pathophysiological changes in OHSS include an increase in capillary permeability caused by an increased expression of Vascular Endothelial Growth Factor (VEGF), a process either triggered exogenously by the use of human Chorionic Gonadotropin (hCG) for ovulation induction/oocyte maturation or endogenous in early pregnancy following COH [1]. This leads to a fluid shift to the third space and the development of ascites, pleural effusion, or even pericardial effusion, while consecutive hemoconcentration/intravascular volume depletion may lead to electrolyte disorders, thromboembolic complications, and organ dysfunction or even failure [1,3]. Therefore, anticoagulation with low-molecular-heparin (LMWH), monitoring of electrolytes and fluid balance are steady pillars of OHSS treatment. Ascites and consecutive abdominal discomfort/distension are often the most bothersome symptoms for the patient. Nevertheless, paracentesis should only be performed when absolutely necessary (e.g., when the patient is in pain or organ function is impaired).

Notably, the use of diuretics is key in the treatment of non-malignant ascites. Naturally, the question arises whether the use of diuretics like furosemide has the potential to improve OHSS treatment. There are concerns that increased hemoconcentration may elevate the risk of thromboembolic complications by lowering the intravascular volume. The reasoning seems to be of a purely theoretical nature, as several authors state different arguments both for and against the use of diuretics in OHSS patients [4,5,6]. In a case report by Bar-Hava and Homburg from 1993, the authors report faster recovery using furosemide in non-hypovolemic patients with severe OHSS (two cases) [7]. To the best of our knowledge, there has never been a scientific investigation on the use of diuretics and associated complications in patients with OHSS. So far, the use of diuretics in the treatment of OHSS is not mentioned in the latest practice guidelines for the prevention and treatment of OHSS by the American Society of Reproductive Medicine (ASRM) and British Fertility Society (BFS)—and therefore not recommended [1,8].

As a tertiary referral center, the Clinical Division of Gynecological Endocrinology and Reproductive Medicine of the Medical University of Vienna treats the majority of cases with OHSS in eastern Austria. The aim of this study was to describe the distribution of OHSS severity, the average duration of treatment, types and frequency of complications associated with antidiuretic treatment regimens in OHSS patients, and predictive factors for paracentesis. Since the use of furosemide in the treatment of OHSS-related ascites was implemented several years ago at our clinic, a secondary goal was to evaluate the outcome with a focus on complications regarding its use.

## 2. Materials and Methods

### 2.1. Patient Population

In this retrospective cohort study, all cases who underwent treatment for OHSS at the Clinical Division of Gynecological Endocrinology and Reproductive Medicine of the Medical University of Vienna, Vienna, Austria, from January 2005 to June 2023 were included (*n* = 502), regardless of whether they received fertility treatment at our facility or at any other health care provider (private and institutional fertility clinics) from in- and outside of Vienna.

OHSS was categorized according to the classification used in the American Society of Reproductive Medicine (ASRM) Practice Committee's clinical practice guideline for OHSS prevention, published in 2024 [1]. Diagnostic evaluation for OHSS was performed by fertility specialists and according to the routine protocol of the Medical University of Vienna, as described in a previous paper from our Division [9]. Patients were hospitalized if at least one of the following criteria was present in addition to abdominal discomfort (including nausea, vomiting, abdominal distension), suggesting at least a moderate grade of OHSS: sonographically detectable free fluid (including ascites and/or pleural effusion), hematocrit > 45%, leucocytosis > 25,000/mm^3^, hypovolemic shock, thromboembolic complications, and/or organ dysfunction. Hospitalization was offered to patients even with mild OHSS if one criterion of all the following three groups shown in Table 1 was met. The main outcome parameter was disease severity, rate and type of complication during OHSS treatment, and treatment duration. Complete data on stimulation protocols, laboratory findings prior to stimulation, and indication for ART were only available for patients receiving COH at our facility. Relevant findings are therefore presented for both patients receiving ART at our facility and all patients treated for OHSS, regardless of where the ART was performed.

The Institutional Review Board (IRB) of the Medical University of Vienna approved the study (IRB Nr.: 1299/2023), which was performed in accordance with the Declaration of Helsinki and the guidelines of Good Scientific Practice, as supported by the Head of the Institute. As this study comprises retrospectively analyzed and anonymized data, the IRB approved the waiver of informed consent.

### 2.2. Standardized OHSS Treatment

The standardized treatment protocol for our division was available to all physicians treating OHSS patients, and adherence to the protocol was mandatory. After initial administration, a complete blood test, including blood count, hematocrit, serum albumin, blood creatinine, creatinine clearance, liver and renal function parameters, as well as coagulation parameters, was obtained. Blood tests, body weight, hydric balance, and abdominal circumference were obtained on a daily basis. The treatment protocol included relative bed rest, volume substitution with 1000 mL lactated isotonic saline solution (0.9% NaCl) infused slowly over a period of 4–6 h once or twice daily, together with furosemide 20–40 mg and, depending on the electrolyte status, an additional 20 mval potassium chloride. Only dydrogesterone or micronized progesterone was used for luteal support, while paracetamol was administered for analgesia. Paracentesis was only performed in patients with severe ascites that caused pain, compromised organ function, or breathing. The assessment of ascites was performed using transvaginal ultrasound; when free fluid exceeded the fundus of the uterus, additional transabdominal ultrasound was performed to assess fluid in the Morison and Koller pouch. Together with clinical examination, ultrasound findings helped guide the decision whether paracentesis was necessary or not [10,11]. Following paracentesis, 20 g albumin was administered intravenously. All patients had to wear full-length venous support stockings and received subcutaneous LMWH in prophylactic dosage of 4000 IU every 24 h. Beginning from January 2008, all patients were treated according to the latest recommendations by the Practice Committee of the American Society for Reproductive Medicine. From 2009 onward, patients with early-onset OHSS received cabergoline 0.5 mg for up to eight consecutive days after oocyte retrieval and, in case of moderate/severe early-onset OHSS, letrozole 2.5–5 mg once daily until estrogen levels fell below 2000 pg/mL as adjuvant therapy [12,13]. According to our treatment protocol, cabergoline and letrozole were not used in patients with late-onset OHSS. Patients were considered recovered and discharged after two consecutive hematocrit levels below 40%, achieving electrolyte balance, normal leucocyte count, and creatinine < 1 mg/dL without therapy, as previously described by Nouri et al. [9].

### 2.3. Parameters Analyzed

Data acquisition was performed using the SAP-based patient management software of the Medical University of Vienna (AKIM Software, Version 7, SAP Software Solutions Austria, Vienna, Austria). The main outcome parameter was the distribution of severity of OHSS. All blood samples and serum parameters were examined in the ISO-certified central laboratory of the General Hospital of Vienna, Austria, using commercially available assays. Enhanced chemiluminescence immunoassay systems were used to determine serum levels of LH, FSH, and estradiol. An enzyme-linked immunosorbent assay was used to determine AMH levels (AMH; DSL Active MIS/AMH assay; Beckman Coulter Inc., Brea, CA, USA). All AMH values before August 23rd 2013 were corrected using the following formula: y = 2.01*x (R^2^ = 0.98). This was due to a complement interference problem of the Beckman AMH ELISA before August 2013, when a dilution protocol had been implemented [14]. Details about the tests applied are provided online at http://www.kimcl.at (accessed on 5 June 2026).

Additionally, data on basic patient characteristics were collected (body mass index (BMI), age, number of previous stimulations, number of previous pregnancies, gravidity). Early-onset OHSS was defined as OHSS that occurred within nine days after the administration of exogenous hCG before oocyte retrieval, while late-onset OHSS—caused by endogenous hCG expression from the initiated pregnancy—is therefore defined as OHSS developing after 10 days [15]. The following parameters on the stimulation protocols were also collected: indication for COH, type of stimulation (long protocol vs. antagonist protocol), type of gonadotropin used for COH (Follicle Stimulating Hormone (FSH) or Human Menopausal Menotropin (HMG)), cumulative dosage of gonadotropins, substance used for ovulation induction (human Chorionic Gonadotropin (hCG) vs. triptorelin), number of retrieved oocytes, fresh-embryo transfer vs. frozen-embryo transfer.

### 2.4. Statistical Analysis

Statistical analyses were conducted using the SPSS software package, version 25.0 (IBM SPSS, Chicago, IL, USA). All continuous and categorical variables are reported as median values (interquartile ranges, IQR) and numbers (%), respectively. To compare categorical variables between the groups, Chi-square or Fisher’s exact tests were used, while Mann–Whitney U tests for independent variables were used to compare continuous variables. Correlations between variables were analyzed using Spearman’s correlation test. A binary logistic regression model was used to evaluate parameters associated with the need for ascites drainage. Univariate analyses were calculated, and all significant parameters were entered into a multivariate model. For these analyses, odds ratios (OR) and 95% confidence intervals (95% CI) are provided. Results were considered statistically significant with *p*-values < 0.05.

## 3. Results

### 3.1. Basic Patient Characteristics

There were 502 cases of in- and outpatient OHSS treatment in 457 individual women. While the vast majority of patients were treated only once (*n* = 422), twenty-seven, six, and two women had to be admitted to the ward twice, three and four times, respectively. 50.2% of patients performed COH at our facility. Notably, complete data sets including laboratory data prior to COH are only available for patients who have undergone ART at our facility. Data from patients who had undergone COH at a private institution were partially incomplete. The median patient age of the whole study population was 31 years (IQR 27–35), with a median BMI of 22.6 kg/m^2^ (IQR 20.8–25.4) at the beginning of the OHSS treatment. While the “in-house” patients showed a median age of 30 years (IQR 26–34), the median BMI was the same. Prior to COH, the median AMH in both groups was 5.7 ng/mL (IQR 3.6–9.1 and 3.6–9.0). In four out of five cases, the antagonist protocol was used to perform COH in both groups. An hCG-containing trigger was used in 79.6% and 81% of stimulations. As expected, the majority of OHSS was early-onset. Table 2 provides further details about basic patient characteristics and OHSS-specific data.

### 3.2. Incidence of OHSS and Disease Severity

**Figure 1:**
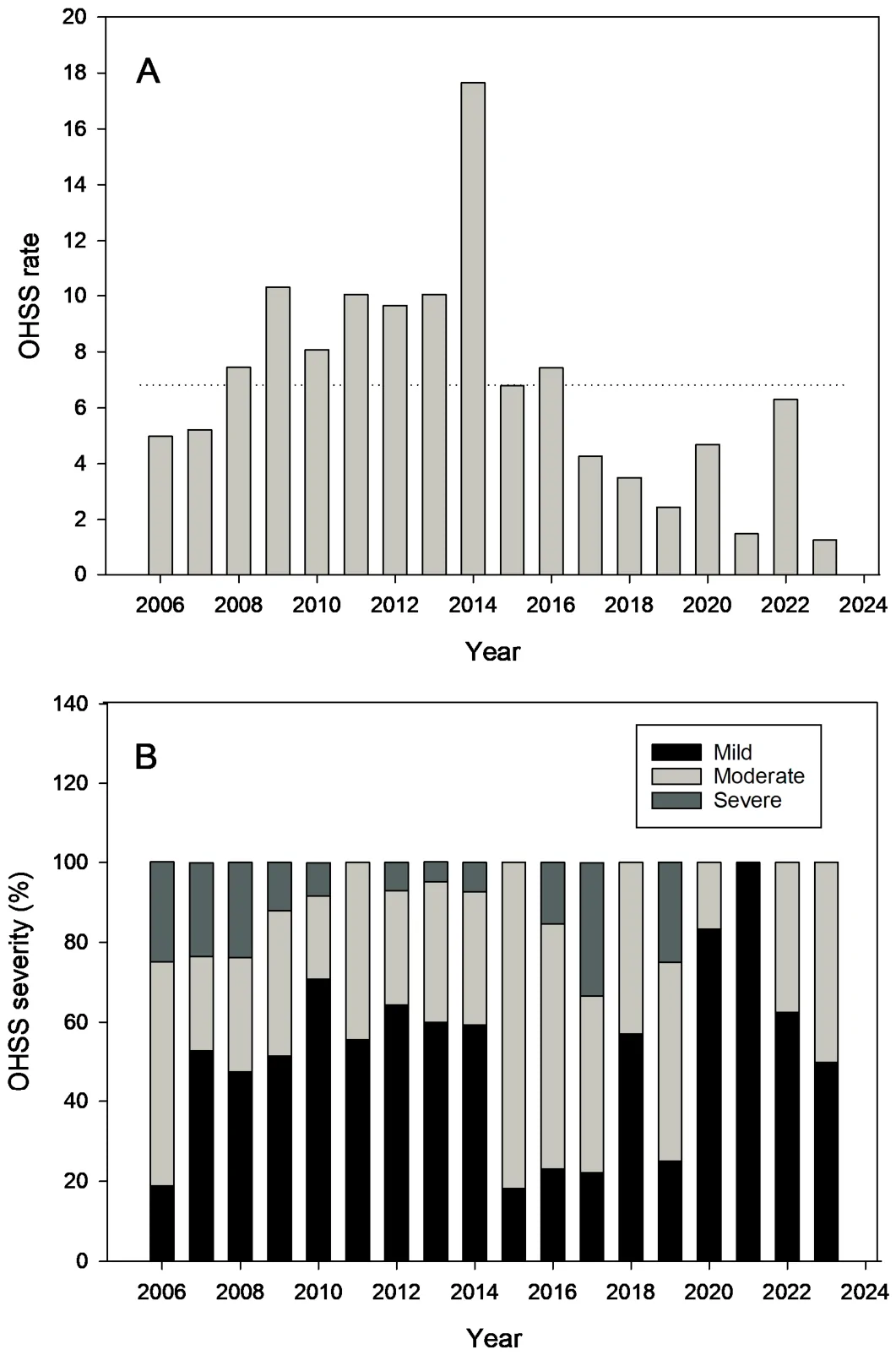
(**A**) Incidence of OHSS in the “in-house” population from 2003 to 2023, dashed line indicating median OHSS rate over the study period, (**B**) distribution of disease severity from 2003 to 2023.

The incidence of OHSS and disease severity was analyzed in the “in-house” population (Figure 1). From 2006 to 2016, the incidence was 8.4% (214/2559), with a wide annual range of incidences, which varied from 5.2% (2017) to 17.6% (2014). From 2015 on, the incidence dropped to 3.2%. A peak was seen in 2022 (6.3%) and the lowest value in 2023 (1.3%). The distribution of disease severity can be seen in Figure 1; annual figures from 2003 to 2023 are provided in Supplement Table S1.

Table 3 shows the distribution of OHSS severity when using hCG-containing and non-hCG-containing triggers for oocyte maturation; no significant association was found (*p* > 0.05. Even when combining mild with moderate and severe with OHSS, no association was found (*p* > 0.05).

### 3.3. OHSS Treatment and Complications

One woman had been referred to our division with already diagnosed deep-vein thrombosis before the start of an OHSS-specific therapy. No new case of thromboembolic complication occurred during the treatment in the whole study population. Eight patients (1.6%) were excluded from the analysis due to largely missing data, since the analog medical records could not be retrieved. Most patients revealed mild or moderate OHSS, whereas only 16% had severe and critical OHSS (see Table 4). The median duration of inpatient treatment was seven days (IQR: 4–10 days). Diuretic treatment was used in 76%. Ascites and pleura drainage was necessary in 28% and 2.0%, respectively.

Average and median cumulative gonadotropin dosages did not significantly differ between the different OHSS grades (*p* = 0.725), see Table 5.

In the in-house group, late-onset OHSS (*p* < 0.001) and a higher initial hematocrit value at the time of admission to the hospital (*p* < 0.001) were associated with the need for ascites drainage (Table 6).

## 4. Discussion

In this retrospective study, we provided a detailed evaluation of OHSS-related treatment and outcome parameters of patients treated at a tertiary referral center. To the best of our knowledge, this study is the first to investigate such a large cohort of patients receiving a standardized treatment of moderate and severe OHSS, including diuretics.

Most importantly, we demonstrated that no additional thromboembolic complication was observed using a standardized protocol including diuretics, as long as adequate intravascular hydration was maintained. Although earlier publications report diuretics to play a beneficial role after hemodilution has been achieved, diuretics are not mentioned in the latest clinical practice guideline for OHSS prevention by the American Society for Reproductive Medicine (ASRM) Practice Committee from 2024 [1,16].

The use of diuretics in OHSS is controversial. Loop diuretics inhibit sodium and chloride reabsorption, leading to increased water excretion, thereby reducing ascites volume. In a 1993 review, Schenker reports diuretics to be ineffective in treating fluid accumulation in the third space, which might lead to the false illusion of adequate renal function due to temporarily increased urine excretion [6]. Delvigne and Rozenberg do not recommend the general use of furosemide either, as its use should be considered only when hemoconcentration with consecutive oliguria persists [5]. In contrast, in 2014, Nouri et al. argued that the use of diuretics helps to prevent renal dysfunction/failure in patients with severe OHSS when combined with adequate intravascular volume substitution and fluid management [9]. The observation made by Bar-Hava and Homburg seems plausible: the use of furosemide can reduce the time to recovery by promoting diuresis, ultimately leading to a reduction of extravascular fluid and therefore relief of symptoms [7]. In a retrospective cohort study by Nouri et al., the average length of inpatient stay for patients with early-onset OHSS was 8 days [17]. In our study, average inpatient treatment lasted 7 days. Therefore, our findings suggest a potential role of furosemide in reducing the duration of inpatient treatment. However, due to the limitations inherent to the study design, these findings should be interpreted with caution and do not allow for generalizable conclusions. Hypokalemia is a frequent and dangerous side effect of loop diuretics, which none of our patients developed. Maintaining normovolemic conditions, observing electrolyte levels on a regular basis, and initiating potassium substitution early in the treatment are therefore crucial for safe use. Our findings further favor the assumption that the use of diuretics, combined with intravenous fluid substitution and electrolyte substitution, can prevent renal complications.

In 2019, Selter et al. published data from a US national database (Nationwide Inpatient Sample—NIS), showing that 2.2% of patients hospitalized for OHSS experienced thromboembolic complications, 1.5% developed acute respiratory distress syndrome (ARDS), 0.9% acute renal failure, and 0.5% needed intubation [18]. In our cohort, only one patient (0.19%) had a thromboembolic complication. Of note, this particular patient had already described pain in the upper limb prior to oocyte retrieval; therefore, this case of thrombosis of the upper limb is more likely an adverse event during COH than a complication of the OHSS treatment itself. A possible explanation for our low number of thromboembolic complications is that the public healthcare system in Austria allows low-threshold inpatient treatment. Neither the hospital stay nor the medication itself has to be paid for directly by the patients, and health care costs are heavily state-subsidized. As a consequence, healthcare providers can more readily offer prophylactic or supportive treatment (e.g., prophylactic anticoagulation), which might not be considered necessary when cost is an issue.

Approximately 30 percent of all patients underwent ascites drainage at our center, a proportion comparable to previous reports. In a retrospective study by Huang et al., 51.7% of patients with moderate or severe OHSS received peritoneal paracentesis [19], while in a study by Nouri et al., 25.4% of patients with severe OHSS were in need of paracentesis [9]. As expected, high hematocrit levels with median values of 42.3% and early-onset OHSS serve as predictive factors for ascites drainage, whereas the diagnosis of PCOS alone does not. A high antral follicle count and AMH are considered the best predictive indicators for developing OHSS [20]. With a median AMH of 5.7 ng/mL, our patients are considered a high-risk population for developing OHSS, whether or not PCOS has been diagnosed. With an anticipated growing demand for fertility preservation among adolescent and young adult oncology patients, the number of women with high AMH levels and, consequently, an increased risk of OHSS undergoing COH may also rise. This may be particularly relevant in patients for whom only a single stimulation cycle is feasible because of the need to initiate cancer treatment without delay. Therefore, such high-risk patients may benefit from alternative fertility preservation strategies, such as ovarian tissue cryopreservation. These approaches enable FP even when there is not enough time for COH or in patients who have not yet reached puberty [21,22].

Our data reveal that the incidence of OHSS declined over the study period, most likely due to a different prophylactic approach to stimulation protocols and technical advantages concerning embryo vitrification (freeze-all policy).

The following study limitations need to be considered: due to the retrospective study design and the heterogeneous documentation of clinical parameters over time, we had to deal with missing values. Many patients were referred to our center from private fertility institutions in and outside of Vienna, and therefore information on the stimulation protocol, trigger of oocyte maturation, cumulative gonadotropin dosages, initial AMH levels, and other important clinical aspects was missing or only reported incompletely. Therefore, we presented relevant findings for both patients receiving ART at our facility and all patients, regardless of whether ART was performed in-house or at a private institution. Owing to the retrospective study design and the absence of an appropriate comparison group, causal conclusions regarding the safety and efficacy of furosemide in OHSS treatment, length of hospitalization, and rate of complications cannot be made, although favorable outcomes have been observed. Although the long study period from 2005 to 2023 enabled the analysis of a large population, several innovations in ovarian stimulation protocols were introduced over the last ten to fifteen years. Individualized stimulation protocols, the substitution of hCG trigger with GnRHa, the implementation of cycle segmentation (“freeze-all” approach), and the administration of dopamine agonists during the luteal phase are the most important developments in OHSS prevention and most certainly the main reason for the observed decrease in OHSS incidence [23]. However, our findings suggest that once OHSS has developed, its clinical course does not differ according to the substance used. Therefore, it can be assumed that GnRHa-only triggers may influence the rate of early-onset OHSS, but not its clinical course once it has developed. A potential source of bias is that furosemide was prescribed more frequently in patients with moderate and severe OHSS than in those with mild OHSS (*p* < 0.001) (Supplement Table S2). A study by Várnagy et al. reported a lower rate of thromboembolic complications in patients receiving low-dose aspirin during COH [24]. We did not assess data on the use of low-dose aspirin and therefore cannot rule out potential confounding bias. Consequently, causal relationships can only be assumed with caution and hypotheses made need to be addressed in further prospective trials.

## 5. Conclusions

In conclusion, our data lend support to the hypothesis that the use of furosemide in the treatment of moderate and severe OHSS can reduce the OHSS-related symptoms, thereby possibly reducing the need for invasive procedures like paracentesis. Moreover, our data suggest that there is no additional risk of developing thromboembolic or renal complications when using diuretics, as long as adequate intravascular hydration and caution are maintained. To the best of our knowledge, this is the first retrospective study to investigate such a large cohort of patients receiving a standardized treatment including diuretics. Further prospective studies focusing on loop diuretics in OHSS treatment are warranted.

## Figures and Tables

**Table 1:** Criteria for hospitalization (modified after Nouri et al. [9]).

(I)	presence of clinically relevant symptoms (nausea, vomiting, abdominal discomfort, dyspnea and tachypnea, subjective decreased urinary output, clinical signs, such as oliguria < 600 mL in 24 h, and/or hypotension)
(II)	electrolyte imbalance, leukocytosis > 20,000/mm^3^, serum creatinine > 1 mg/dL, and/or hematocrit > 45%; and
(III)	sonographically detectable free fluid (ascites and/or pleural effusion), and a significant increase in ovarian diameter (>12 cm).

**Table 2:** Basic patient characteristics.

	Whole Study Population (*n* = 502)		Patients Who Underwent COH in-House (*n* = 252)	
		Missing Data		Missing Data
Age (years) ^1^	31 (27; 35)	0	30 (26; 34)	0
BMI before ovarian hyperstimulation (kg/m^2^) ^1^	22.6 (20.8; 25.4)	149	22.6 (20.9; 25.4)	0
Gravidity ^1^	0 (0; 1)	94	0 (0; 1)	0
Parity ^1^	0 (0; 1)	101	0 (0; 0)	1
Polycystic ovary syndrome ^2^	94 (28.1)	168	70 (27.8)	0
AMH before stimulation (ng/mL) ^1^	5.7 (3.6; 9.1)	344	5.7 (3.6; 9.0)	97
TSH before stimulation (µIU/mL) ^1^	1.62 (1.10; 2.08)	269	1.63 (1.11; 2.08)	22
Number of retrieved oocytes ^1^	16 (11; 20)	152	15 (11; 18)	8
Total FSH dose used (units) ^1^	1800 (1450; 2200)	254	1800 (1450; 2250)	18
Long protocol ^2^	64 (20.9)	196	48 (19.0)	0
hCG trigger ^2^	227 (79.6)	218	204 (81.0)	0
Early-onset OHSS ^2^	357 (71.7)	0	212 (84.1)	0

Data are provided as ^1^ median (IQR) or ^2^ *n* (%); the percentage refers to the valid number of patients (excluding missing values).

**Table 3:** Trigger substance and OHSS severity.

	Trigger Containing hCG	Trigger Not Containing hCG
Mild OHSS	114	27
Moderate OHSS	82	23
Severe OHSS	30	7
Critical OHSS	1	0

Data on trigger substance available for some patients receiving ART outside our facility.

**Table 4:** OHSS treatment characteristics.

		Whole Study Population (*n* = 494)	Patients Who Underwent COH in-House (*n* = 252)
OHSS severity ^2^	Mild	221 (44.7)	128 (50.8)
	Moderate	192 (38.9)	95 (37.7)
	Severe	76 (15.4)	29 (11.5)
	Critical	1 (0.2)	0
Duration of inpatient treatment (days) ^1^		7 (4; 10)	7 (4; 10)
Use of diuretics ^2^		374 (75.7)	173 (68.7)
Use of “diuretics perfusor” ^2^		88 (17.8)	29 (11.5)
Use of HAES ^2^		37 (7.5)	16 (6.3)
Ascites drainage		138 (27.9)	49 (19.4)
Pleura drainage		12 (2.0)	2 (0.8)

Data are provided as ^1^ median (IQR) or ^2^ *n* (%); the percentage refers to the valid number of patients (excluding missing values).

**Table 5:** Cumulative gonadotropin (Gn) dosage and OHSS severity.

	Median Gn Dosage (I.E.)	Average Gn Dosage (I.E.)
Mild OHSS	1800	1921
Moderate OHSS	1725	2006
Severe OHSS	1875	1820
Critical OHSS	Missing value	

**Table 6:** Predictive factors for ascites drainage. Results of a binary logistic regression model (in-house patients with complete data set only). Statistically signicant results are in italics.

	Ascites Drainage (*n* = 49)	No Ascites Drainage (*n* = 203)	OR (95% CI)	*p*	Adj OR (95%CI)	*p*
Age (years)	30 (26; 33)	30 (26; 34)	1.010 (0.954; 1.070)	0.729	-	-
BMI (kg/m^2^)	22.2 (20.9; 24.4)	22.7 (20.9; 25.6)	1.018 (0.946; 1.097)	0.630	-	-
PCOS	16 (32.7)	54 (26.6)	1.338 (0.682; 2.623)	0.397	-	-
Use of hCG	43 (87.8)	161 (79.3)	1.870 (0.746; 4.687)	0.182	-	-
Early-onset OHSS	32 (65.3)	180 (88.7)	0.241 (0.116; 0.500)	*<0.001*	0.260 (0.119; 0.570)	*<0.001*
Abdominal circumference at admission (cm)	85 (80; 94)	87 (80; 94)	0.993 (0.962; 1.026)	0.686	-	-
Hematocrit at admission (%)	42.3 (38.0; 45.0)	38.0 (35.8; 40.7)	1.2121 (1.118; 1.315)	*<0.001*	1.202 (1.107; 1.306)	*<0.001*

## Data Availability

The datasets generated and/or analyzed in the current study are not publicly available, since the dataset will be used for other retrospective analyses. The data are available from the corresponding author upon reasonable request.

## Supplementary Materials

The following supporting information can be downloaded at: https://www.mdpi.com/article/10.3390/jcm15166468/s1, Table S1: Annual distribution of disease severity from 2003 to 2023 (“in-house” population); Table S2: Diuretic administration based on OHSS severity.

## Abbreviations

AFC	Antral Follicle Count
AMH	Anti-Müllerian Hormone
ART	Assisted Reproductive Technologies
ASRM	American Society of Reproductive Medicine
ATE	Arterial Thrombotic Event
COH	Controlled Ovarian Hyperstimulation
FP	Fertility Preservation
FSH	Follicle Stimulating Hormone
Gn	Gonadotropine
GnRHa	Gonadotropin-Releasing Hormone Agonists
hCG	Human Chorionic Gonadotropin
IRB	Institutional Review Board
IU	International Units
IVF	In Vitro Fertilization
LH	Luteinizing Hormone
LMWH	Low molecular Weight Heparin
OHSS	Ovarian Hyperstimulation Syndrome
PCOS	Polycystic Ovarian Syndrome
TSH	Thyroid Stimulating Hormone
VEGF	Vascular Endothelial Growth Factor
VTE	Venous Thromboembolism
