# Supplementary material for: Clinical Management of Ovarian Hyperstimulation Syndrome with Furosemide at a Tertiary Referral Center—A Retrospective Data Analysis
Source: J Clin Med. 2026 Aug 21;15(16):6468. doi: 10.3390/jcm15166468 (PMC13514558; doi:10.3390/jcm15166468)
Supplement: Supplementary file 1 [file jcm-15-06468-s001.zip › jcm-4439934-supplementary.pdf]

| **Supplement Table S1:** Annual distribution of disease severity from 2003 to 2023 (“in-house” population) | | | | | | | | | |
|---|---|---|---|---|---|---|---|---|---|
| Year | n stimulation cycles in-house | n OHSS in-house | % OHSS | Mild | % | Moderate | % | Severe | % |
| 2006 | 321 | 16 | 5,0 | 3 | 18,8 | 9 | 56,3 | 4 | 25 |
| 2007 | 326 | 17 | 5,2 | 9 | 52,9 | 4 | 23,5 | 4 | 23,5 |
| 2008 | 282 | 21 | 7,4 | 10 | 47,6 | 6 | 28,6 | 5 | 23,8 |
| 2009 | 320 | 33 | 10,3 | 17 | 51,5 | 12 | 36,4 | 4 | 12,1 |
| 2010 | 297 | 24 | 8,1 | 17 | 70,8 | 5 | 20,8 | 2 | 8,3 |
| 2011 | 179 | 18 | 10,1 | 10 | 55,6 | 8 | 44,4 | 0 | 0 |
| 2012 | 145 | 14 | 9,7 | 9 | 64,3 | 4 | 28,6 | 1 | 7,1 |
| 2013 | 199 | 20 | 10,1 | 12 | 60 | 7 | 35,1 | 1 | 5 |
| 2014 | 153 | 27 | 17,6 | 16 | 59,3 | 9 | 33,3 | 2 | 7,4 |
| 2015 | 162 | 11 | 6,8 | 2 | 18,2 | 9 | 81,8 | 0 | 0 |
| 2016 | 175 | 13 | 7,4 | 3 | 23,1 | 8 | 61,5 | 2 | 15,4 |
| 2017 | 211 | 9 | 4,3 | 2 | 22,2 | 4 | 44,4 | 3 | 33,3 |
| 2018 | 200 | 7 | 3,5 | 4 | 57,1 | 3 | 42,9 | 0 | 0 |
| 2019 | 164 | 4 | 2,4 | 1 | 25 | 2 | 50 | 1 | 25 |
| 2020 | 128 | 6 | 4,7 | 5 | 83,3 | 1 | 16,7 | 0 | 0 |
| *A single case of critical OHSS is not included in this table* | | | | | | | | | |

| **Supplement Table S2:** Diuretic administration based on OHSS severity | | | | |
|---|---|---|---|---|
| | | OHSS severity | | |
| | | Mild + Moderate OHSS | Severe + Critical OHSS | total |
| Administration of diuretics | No | 118 | 2 | 120 |
| | Yes | 297 | 77 | 374 |
| total | | 415 | 79 | 494 |
| *Patients with severe and critical OHSS were prescribed furosemide at a statistically significant higher rate (p<0.001).* | | | | |
